# Reactive oxygen species mediate TNFR1 increase after TRPV1 activation in mouse DRG neurons

**DOI:** 10.1186/1744-8069-5-31

**Published:** 2009-06-17

**Authors:** Fei Ma, Liping Zhang, Karin N Westlund

**Affiliations:** 1 Department of Physiology, University of Kentucky, Lexington, Kentucky 40536-0298, USA

## Abstract

**Background:**

Transient receptor potential vanilloid subtype 1 (TRPV1) is activated by low pH/protons and is well known to be involved in hyperalgesia during inflammation. Tumor necrosis factor α (TNF-α), a proinflammatory cytokine, is involved in nociceptive responses causing hyperalgesia through TNF receptor type 1 (TNFR1) activation. Reactive oxygen species (ROS) production is also prominently increased in inflamed tissue. The present study investigated TNFR1 receptors in primary cultured mouse dorsal root ganglion (DRG) neurons after TRPV1 activation and the involvement of ROS. C57BL/6 mice, both TRPV1 knockout and wild type, were used for immunofluorescent and live cell imaging. The L4 and L5 DRGs were dissected bilaterally and cultured overnight. TRPV1 was stimulated with capsaicin or its potent analog, resiniferatoxin. ROS production was measured with live cell imaging and TNFR1 was detected with immunofluorescence in DRG primary cultures. The TRPV1 knockout mice, TRPV1 antagonist, capsazepine, and ROS scavenger, N-tert-Butyl-α-phenylnitrone (PBN), were employed to explore the functional relationship among TRPV1, ROS and TNFR1 in these studies.

**Results:**

The results demonstrate that TRPV1 activation increases TNFR1 receptors and ROS generation in primary cultures of mouse DRG neurons. Activated increases in TNFR1 receptors and ROS production are absent in TRPV1 deficient mice. The PBN blocks increases in TNFR1 and ROS production induced by capsaicin/resiniferatoxin.

**Conclusion:**

TRPV1 activation increases TNFR1 in cultured mouse DRG neurons through a ROS signaling pathway, a novel sensitization mechanism in DRG neurons.

## Background

Inflammation comes with sensitization of specialized sensory neurons that comprise the nociceptive pathway, leading to enhanced pain sensation in response to both noxious and non-noxious stimuli [1]. The transient receptor potential vanilloid 1 (TRPV1) participates in the development of chemical and thermal hyperalgesia in inflammation, through the action of low pH, heat and inflammatory mediators [2]. The most striking feature of TRPV1 knockout mice is the virtual absence of thermal hypersensitivity in the setting of inflammation [3-6]. Inflammation causes the release of inflammatory mediators which lower the threshold of heat stimulation, a process known as heat hyperalgesia depending on TRPV1 activation [7].

Tumor necrosis factor α (TNF-α) is prominently released during inflammation and plays an important role in hyperalgesia in inflammation. Application of TNF-α enhances calcium currents increasing neuron sensitivity to the neurotoxin capsaicin in cultures of sensory neurons, and anti-TNF therapy produces a profound analgesia [8-11]. TNF-α acts on two distinct subtypes of receptors, TNF receptor type 1 (TNFR1, p55) and TNF receptor type 2 (TNFR2, p75) [12]. TNFR1 but not TNFR2 neutralizing antibodies, as well as antisense RNA against TNFR1, can reduce experimentally induced hyperalgesia [13,14]. TNFR1 immunoreactivity (IR) has been reported in DRG neurons [15,16]. TNFR1 is increased in nociceptive DRG neurons and primary afferents by intraperitoneal lipopolysaccharide and after nerve injury in animal models, and in plasma of patients with pain syndromes, supporting an important role for TNFR1 in nociception [17-19].

While reactive oxygen species (ROS) are primarily important for regulating many metabolic cellular activities, excess ROS have been implicated in neuropathic and inflammatory pain [20-23]. The transient but dramatic analgesic effect of free-radical scavengers in pain models suggests that ROS are critically involved in the generation of pain. Some research on the signaling pathways has been directed towards documenting a role for ROS in cell death and the induction of cell proliferation [24-26]. The effects of ROS may be mediated by reversible effects on intracellular proteins which lead to alterations in intracellular signaling pathways. Effects of ROS on aberrant signaling pathways in the nervous system remain to be determined.

Although some reports show that TNF-α sensitizes TRPV1, we are interested in the role that TRPV1 activation might have on TNFR1 as the drive for maintaining nociceptor sensitization. The present study investigated changes in TNFR1 in response to TRPV1 activation in primary DRG neuronal cultures from both TRPV1 knockout and wild type mice while monitoring involvement of ROS as a potential drive in this signaling pathway. Primary DRG neurons were dissected from wild type and TRPV1 knockout C57BL/6 mice. After overnight incubation, DRG cultures were challenged with either capsaicin or resiniferatoxin.

## Results

### Capsaicin induces TNFR1 staining increases in DRG neurons from wild type mice

**Figure 1:**
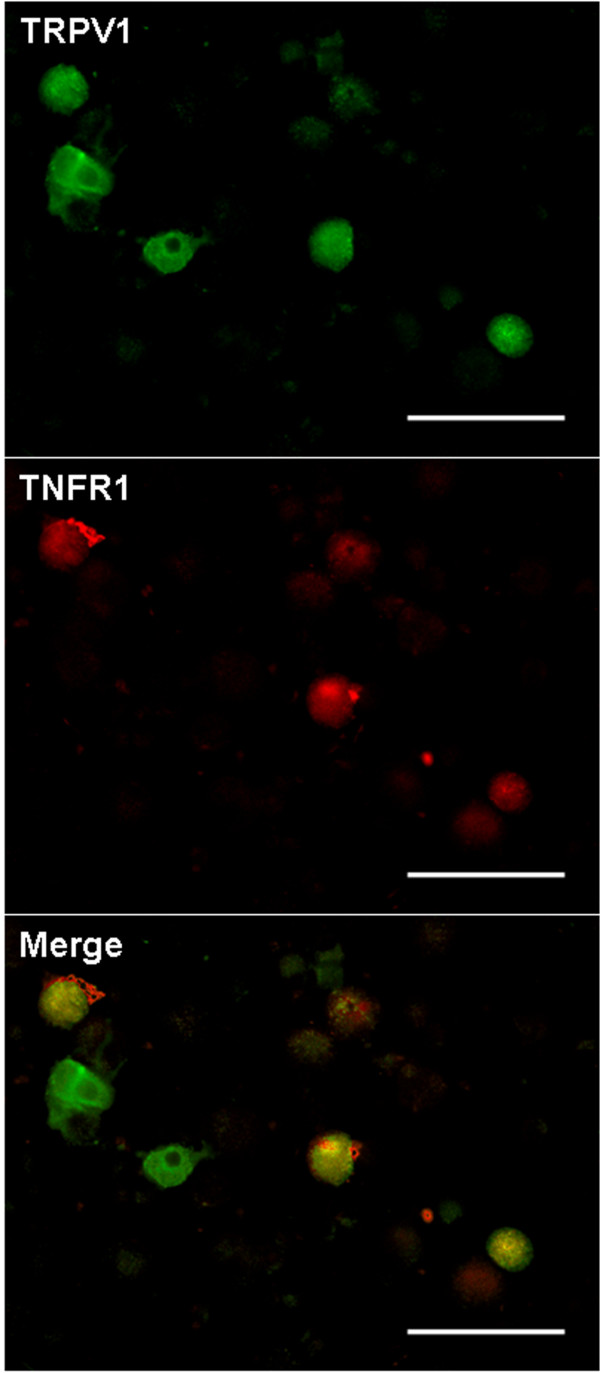
**TRPV1 and TNFR1 in primary cultures of mouse DRG neurons**. Photomicrographs show green TRPV1 immunofluorescence and red TNFR1 immunofluorescence in DRG neurons cultured overnight and then fixed for double staining. Merge: Double labeling of TRPV1 and TNFR1 immunostaining (yellow) indicates that TRPV1 and TNFR1 are co-localized in cultured mouse DRG neurons. Scale Bars: 100 μm.

**Figure 2:**
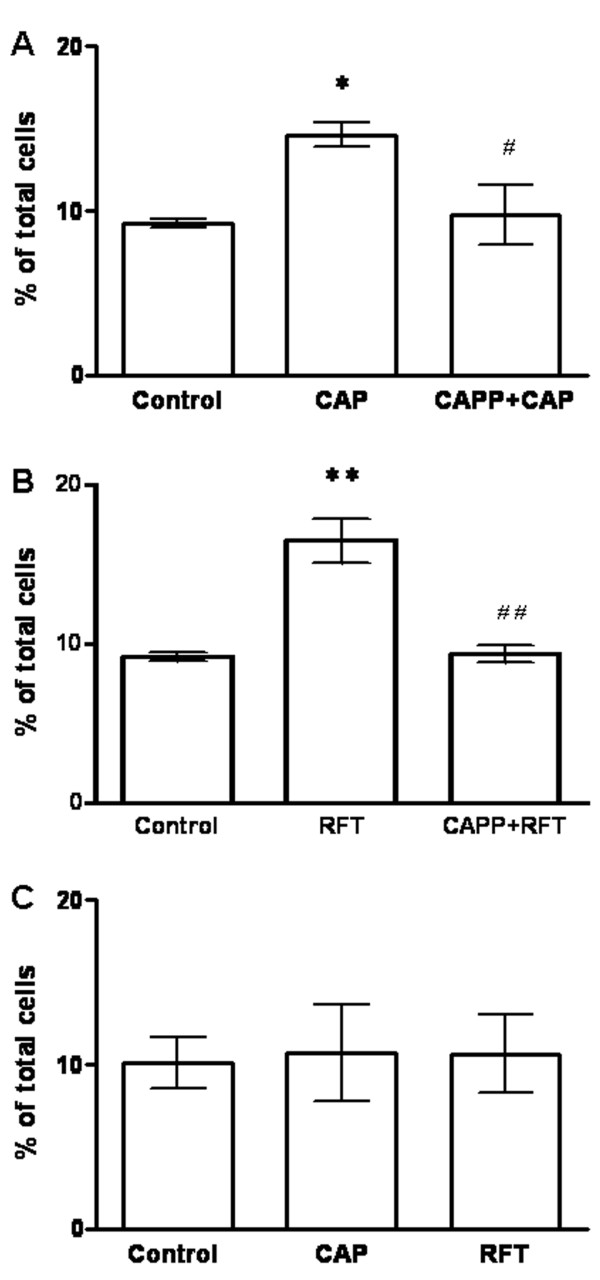
**Capsaicin or resiniferatoxin activation of TRPV1 increases TNFR1 staining in cultured mouse DRG neurons**. Histograms show the percentage of immunoreactive cells in DRG neuronal cultures. (A) Capsaicin (CAP) significantly increases TNFR1 staining (*P < 0.05) and capsazepine (CAPP) blocks TNFR1 staining increases induced by capsaicin (^#^*P *< 0.05 compared to capsaicin alone). (B) Resiniferatoxin (RFT) significantly increases TNFR1 (**P < 0.01) and capsazepine blocks the TNFR1 increase induced by resiniferatoxin (^##^*P *< 0.01 compared to resiniferatoxin alone). (C) There is no change of TNFR1 in DRG neurons from TRPV1 knockout mice after capsaicin or resiniferatoxin stimulation.

In untreated control primary cultures (24 hr) of DRG neurons from wild type mice, 22.82 ± 1.68% of the neurons stained for TRPV1, and 9.24 ± 0.27% stained for TNFR1 (Table 1). Photomicrographs shown in Figure 1 illustrate the green immunofluorescent staining for TRPV1 and red immunofluorescence identifying TNFR1. In order to explore expression of TNFR1 in mouse DRG neurons after capsaicin stimulation, we first examined the co-localization of TRPV1 with TNFR1. Color combination (yellow) microscopy indicated co-localization of TRPV1 and TNFR1 in some mouse DRG neurons. Dual staining for TRPV1 and TNFR1 was observed in 41.04 ± 3.94% of TRPV1 immunostaining positive neurons (Table 1, Fig. 1 Merge) (n = 592). Staining for TRPV1 and TNFR1 were both observed primarily in small size neurons. Both capsaicin (1 μM, 20 min) and resiniferatoxin (200 nM, 20 min) treated groups had significantly increased TNFR1 staining (capsaicin treated group: 14.62 ± 0.72%, n = 431, *P *< 0.05; resiniferatoxin treated group: 16.5 ± 1.37%, n = 622, *P *< 0.01), compared with the untreated group (Fig. 2A, B). There was an increase in the percentage of neurons showing dual staining for TNFR1 and TRPV1 after treatment with capsaicin or resiniferatoxin. In the capsaicin treated group, TNFR1 was identified in 52.89 ± 2.89% of neurons with TRPV1 immunoreactivity (*P *< 0.05 compared with the untreated group), and TNFR1 was found in 73.63 ± 3.54% of TRPV1 neurons in the resiniferatoxin treated group (*P *< 0.01 compared with the untreated group; *P *< 0.05 compared with the capsaicin treated group). This indicates that primary mouse DRG neurons are more responsive to potent resiniferatoxin as expected (Table 1). However, the TRPV1 staining was not changed after capsaicin or resiniferatoxin stimulation (capsaicin treated group: 27.91 ± 2.58%; resiniferatoxin treated group: 23.19 ± 2.15% (*P *> 0.05 for both compared with the untreated group 22.82 ± 1.68%)) (Table 1).

**Table 1:** Percentage of TNFR1 and TRPV1 positive neurons in primary cultures of DRG neurons from wild type mice

	**TNFR1**		**TRPV1**		**Total neurons**	**TNFR1** **(% TRPV1)**
			
	**Positive**	**% of total**	**Positive**	**% of total**		
**Untreated**	54	9.24 ± 0.27	128	22.82 ± 1.69	592	41.04 ± 3.94
**Capsaicin**	60	14.62 ± 0.72	113	27.91 ± 2.58	431	52.89 ± 2.89
**Resiniferatoxin**	100	16.5 ± 1.37	149	23.19 ± 2.15	622	73.63 ± 3.54

### Capsaicin induced TNFR1 increase is absent in DRG neurons of TRPV1 deficient mice

The TNFR1 staining increase after capsaicin (1 μM, 20 min) or resiniferatoxin (200 nM, 20 min) stimulation was examined in primary cultures of DRG neurons from TRPV1 knockout mice. No significant change was found (capsaicin treated group: 10.71 ± 2.93%; resiniferatoxin treated group: 10.64 ± 2.36% compared with untreated group: 10.09 ± 1.56%) (Fig. 2C). Immunocytochemical staining results also confirmed that TRPV1 staining was absent in cultured DRG neurons of TRPV1 deficient mice in this study.

### Capsazepine blocks capsaicin induced TNFR1 increase in DRG neurons from wild type mice

Capsazepine, a competitive TRPV1 antagonist, was used to determine if capsaicin or resiniferatoxin induced TNFR1 staining in cultured DRG neurons from wild type mice is mediated through TRPV1 receptor activation. The results showed that capsazepine (10 μM, 10 min) blocked TNFR1 increases induced by capsaicin (1 μM, 20 min) or resiniferatoxin (200 nM, 20 min) respectively (Fig. 2A, B) (capsazepine plus capsaicin treated group: 9.74 ± 1.85%, n = 522, *P *< 0.05 compared with the capsaicin treated group: 14.62 ± 0.72%; capsazepine plus resiniferatoxin treated group: 9.34 ± 0.53%, n = 393, *P *< 0.01 compared with resiniferatoxin treated group: 16.5 ± 1.37%).

### PBN blocks capsaicin induced TNFR1 increase in DRG neurons of wild type mice

**Figure 3:**
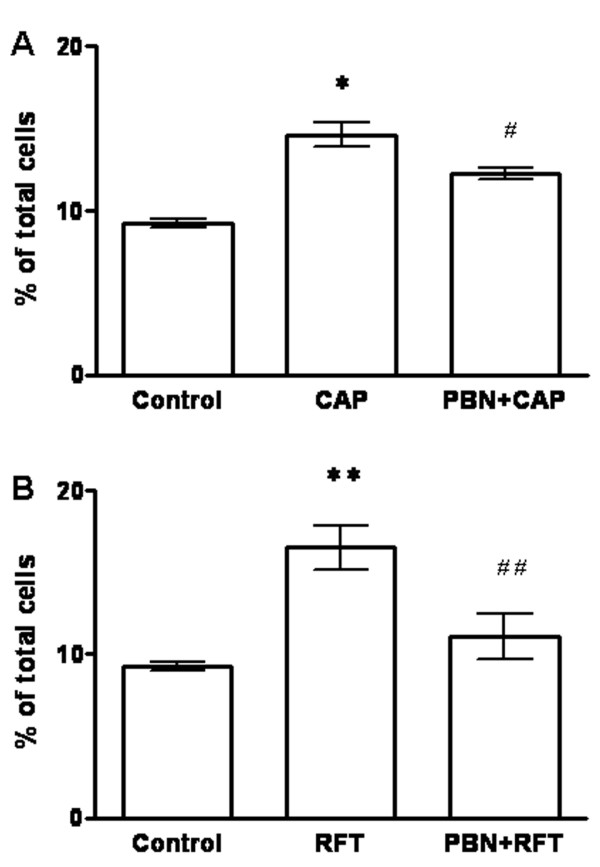
**The ROS scavenger PBN blocks TNFR1 increases in cultured mouse DRG neurons induced by capsaicin or resiniferatoxin**. Histograms showing the percentage of TNFR1 immunoreactive neurons in DRG neuronal cultures. (A) Capsaicin significantly increases TNFR1 staining in DRG neurons (*P < 0.05), and PBN blocks the TNFR1 increase induced by capsaicin (^#^*P *< 0.05 compared to capsaicin). (B) Resiniferatoxin significantly increases the TNFR1 increase in DRG neurons (**P < 0.01). PBN blocks the TNFR1 increase induced by resiniferatoxin (^##^*P *< 0.01 compared to resiniferatoxin). Results indicate that capsaicin and resiniferatoxin increase TNFR1 in DRG neurons through a ROS signaling pathway.

Pretreatment with PBN (2 mM, 90 min) of cultured DRG neurons from wild type mice blocked the TNFR1 increases induced by capsaicin (1 μM) or resiniferatoxin (200 nM) (Fig. 3). TNFR1 staining in DRG neurons in the PBN plus capsaicin treated group (12.28 ± 0.36%, n = 395, *P *< 0.05) was significantly decreased compared with the capsaicin treated group (14.62 ± 0.72%) (Fig. 3A). Significantly decreased TNFR1 staining was also found in DRG neurons of the PBN plus resiniferatoxin treated group (11.08 ± 1.39%, n = 534, *P *< 0.01) compared that with resiniferatoxin treated group (16.5 ± 1.37%) (Fig. 3B).

### PBN blocks TRPV1 activated increases in ROS production in DRG neurons of wild type mice

**Figure 4:**
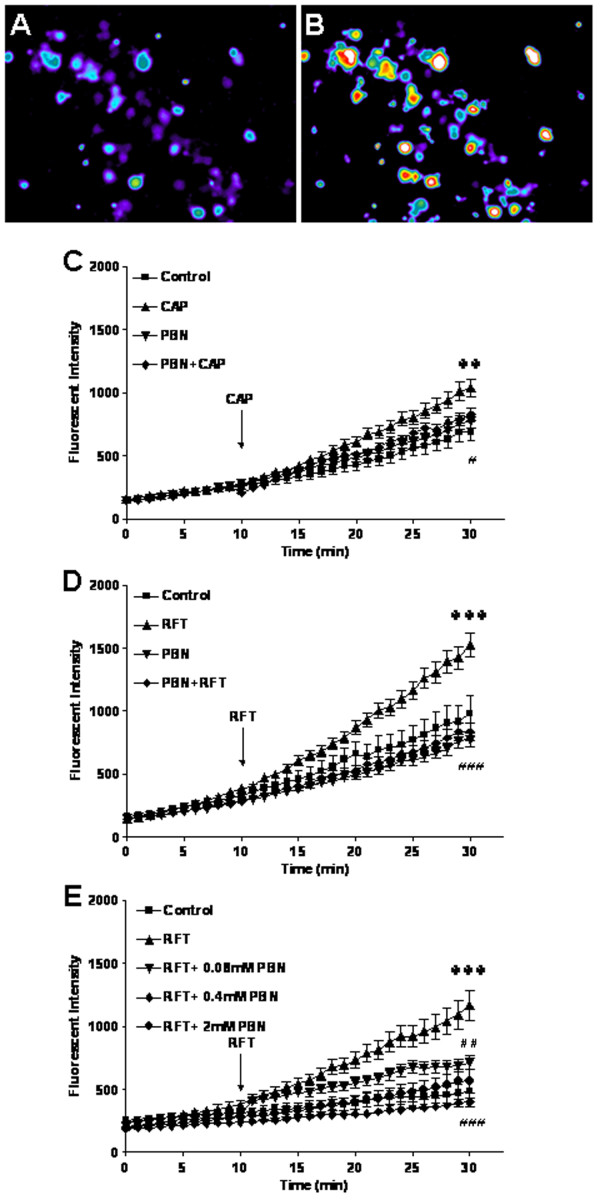
**Capsaicin or resiniferatoxin increases ROS production in mouse primary DRG cultures**. (A) Photomicrographs of ROS in mouse DRG in primary cultures at baseline. (B) ROS production increases after resiniferatoxin stimulation. The effects are indicated by fluorescent dye intensity changes captured with epifluorescent microscopy and quantified with MetaMorphe image analysis. (C) Capsaicin increases ROS production at 30 minutes (**P < 0.01 compared to control) and ROS scavenger PBN (2 mM) blocks capsaicin induced ROS production at 30 minutes (^#^*P *< 0.05 compared to capsaicin). (D) Resiniferatoxin increases ROS production at 30 minutes (***P < 0.001 compared to control) and PBN (2 mM) blocks resiniferatoxin induced ROS production at 30 minutes (^###^*P *< 0.001 compared to resiniferatoxin). (E) PBN also significantly blocks ROS production at two lower doses (0.4 mM (***P < 0.001 compared to resiniferatoxin) and 0.08 mM (**P < 0.01 compared to resiniferatoxin)).

Experiments were performed to analyze ROS production after capsaicin treatment in cultured DRG neurons from wild type mice. N-tert-butyl-α-phenylnitrone (PBN) is an electron spin trap with high avidity for free radical species and functions as an antioxidant in many biological systems. PBN was used to block ROS production after TRPV1 activation with capsaicin. The color intensity increases in the microscope image indicate that oxidized derivative DCF is accumulating in cells (Fig. 4A, B). The DCF accumulation indicates that ROS production is increasing. Although there was a slow, steady increase of ROS production in all neurons in normal physiological experimental solution due to spontaneous oxidation of the dye over time, ROS production was significantly increased in cultured DRG neurons after bath application of capsaicin (1 μM) or resiniferatoxin (200 nM) compared with the untreated neurons. Represented with numerical data at the 30 minute time point, the ROS fluorescent intensity for the capsaicin treated group (1037.57 ± 72.15 arbitrary units, n = 117, *P *< 0.01) was increased compared to the untreated group (693.24 ± 75.84, n = 49, *P *< 0.01); and ROS was increased in the resiniferatoxin treated group (1524.56 ± 97.73, n = 104, *P *< 0.001) compared with the untreated group (980.61 ± 140.60, n = 80, *P *< 0.001) (Fig. 4C, D). The PBN (2 mM) added 90 min prior to start of recording abolished ROS production induced by capsaicin (1 μM) or resiniferatoxin (200 nM) (PBN plus capsaicin treated group: 829.22 ± 51.22, n = 57, *P *< 0.05 compared with capsaicin treated group; PBN plus resiniferatoxin treated group: 835.25 ± 68.83, n = 114, *P *< 0.001 compared with the resiniferatoxin treated group) (Fig. 4C, D). ROS production parallels that of untreated control cultures when treated with PBN only under the same conditions (2 mM for 2 hrs) (Fig. 4C, D). Dose response studies (Fig. 4E) found that two lower doses of PBN (0.08, 0.4 mM) were also effective in significantly reducing the effect of resiniferatoxin at 30 min (712.85 ± 53.71 for the 0.08 mM dose of PBN plus resiniferatoxin, n = 24, *P *< 0.01 compared with resiniferatoxin; 399.55 ± 35.44 for the 0.4 mM dose of PBN plus resiniferatoxin, n = 28, *P *< 0.001 compared with resiniferatoxin).

### Capsaicin increases ROS production in DRG neurons of wild type mice

**Figure 5:**
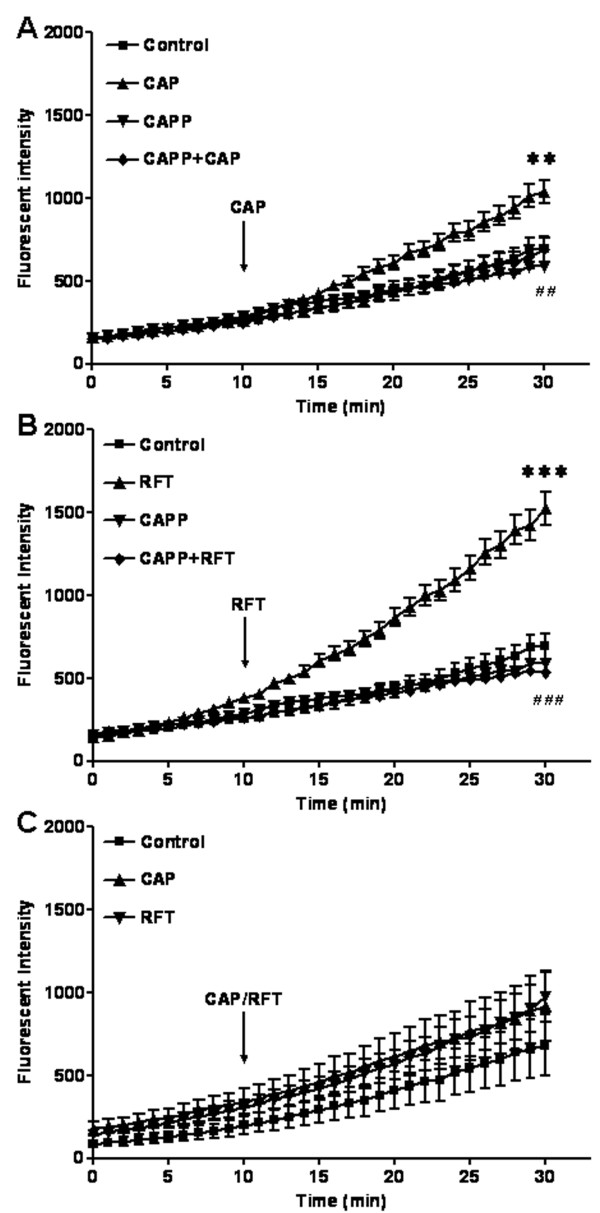
**Capsaicin or resiniferatoxin induces ROS production in cultured mouse DRG neurons through TRPV1 activation**. (A) Capsaicin significantly increases ROS production and capsazepine blocks capsaicin induced ROS production (30 minutes: ^##^*P *< 0.01 compared to capsaicin). (B) Resiniferatoxin significantly increases ROS production and resiniferatoxin blocks capsaicin induced ROS production (30 minutes: ^###^*P *< 0.001 compared to resiniferatoxin). (C) There is no change in ROS production in DRG neurons from TRPV1 deficient mice after capsaicin or resiniferatoxin stimulation.

Experiments were performed in cultured DRG neurons from wild type and TRPV1 knockout mice to determine ROS production changes after capsaicin or resiniferatoxin stimulation. In the experiment with wild type mouse DRG neurons, capsazepine (10 μM) added 10 min prior to start of recording blocked both capsaicin (1 μM) and resiniferatoxin (200 nM) induced increases in ROS production. Values represent the fluorescent dye intensity at the 30 minute time point (capsazepine plus capsaicin treated group: 690.56 ± 73.54, n = 47, *P *< 0.01 compared with capsaicin treated group: 1037.57 ± 72.15; capsazepine plus resiniferatoxin treated group: 535.53 ± 25.46, n = 115, *P *< 0.001 compared with resiniferatoxin treated group: 1524.56 ± 97.73) (Fig. 5A, B). No changes in ROS production were detected in DRG neurons from TRPV1 deficient mice (capsaicin treated group: 916.16 ± 105.09; resiniferatoxin treated group: 975.45 ± 78.10 compared with untreated group: 683.4 ± 92.15) (Fig. 5C).

## Discussion

The present study demonstrates that capsaicin increased both the TNFR1 staining and ROS production in cultured DRG neurons from wild type mice through TRPV1 activation. These phenomena did not occur in TRPV1 deficient mice. ROS scavenger PBN and TRPV1 antagonist, capsazepine, blocked both the TNFR1 and ROS increases. We conclude that TRPV1 activation increases TNFR1 through a signaling pathway driven by ROS generation.

### Regulation of TNFR1 by capsaicin

Two subtypes of TNF-α receptors, TNFR1 and TNFR2, are expressed in peripheral nerves [16]. TNFR1 is expressed in dorsal root ganglion neurons and plays a potential role in nociception [13]. In present experiment, TNFR1 is positively immunostained in 10% of mouse DRG neurons and almost all of which were small size neurons which suggests a potential role of TNFR1 in transmission of nociception. TNFR1 in mouse DRG neurons is detected after sciatic nerve injury but not in control mice [27]. The process of dissociating and culturing DRG neurons apparently increases expression in the cells somewhat since TNFR1 is not found in normal mouse DRG tissue sections. In rat inflammation or nerve injury models, increases in TNFR1 expression are reported by several other researchers [15,17,28-30]. Although there are reports that TNF-α increases TRPV1 expression through TNF-α receptors and TRPV1 induced activity is enhanced by TNF-α administration [31-33], it would be interesting to know if they can reciprocally regulate one another. The interactive relationship of these two types of receptors will be the target of further investigation since the present study suggests TRPV1 activation is inducing TNFR1 increases and/or activation. In the present study using mouse DRG neuronal cultures, TNFR1 was significantly increased after stimulation with capsaicin/resiniferatoxin. The responses to capsaicin and resiniferatoxin were different since resiniferatoxin is a more robust agonist for TRPV1. This result is supported by calcium influx experiments where resiniferatoxin produces a stronger calcium increase [3]. To confirm that TNFR1 increases induced by capsaicin are mediated through TRPV1, TRPV1 knockout mice were employed and additional experiments using capsazepine were also performed. The results of both these experiments showed that capsaicin/resiniferatoxin induced increases in TNFR1 in small size DRG neurons co-expressing TRPV1, and thus are likely to be nociceptors. This novel finding of capsaicin induced TNFR1 increases in cultured mouse DRG neurons has not been reported by other investigators. The result is in agreement with those in a rat inflammation model [13]. In CFA arthritis models *in vivo*, upregulation of TNFR1 receptors is detected in peripheral joint tissue, DRG neurons, and small fiber bundles within the root entry zone and fibers bordering the trigeminal and spinal cord dorsal horn [28,34,35].

During inflammation, high levels of H^+ ^are released from injured tissue and are important mediators activating TRPV1. In fact, there are several other mediators activating TRPV1 such as anandamide and high temperature (43°C) that are present in inflamed tissue targeted by sensory nerve terminals. Thermal hyperalgesia and mechanical allodynia are triggered by TRPV1 over-activation and plastic changes occur in neurons with influxes of ions such as calcium and sodium. One of the changes we find is increasing TNFR1 in TRPV1 stained neurons which would allow neurons to receive information presented in inflammatory conditions of which TNF-α plays an important role. The present study demonstrates the mechanistic linkage between TNFR1 and TRPV1 activation. TNF-α is produced by satellite cells in DRG, Schwann cells in the peripheral nervous system and local macrophage cells which increase TNF-α release from peripheral tissue attracting considerable attention in nociceptive sensation and inflammatory cell invasion during inflammation. TNF-α mRNA increases are observed 24 hours after periganglionic injections of complete Freund's adjuvant [36]. Anti-TNF therapy used in treatment of rheumatoid arthritis produces profound analgesia [8,11], indicating that TNF-α and its receptors may be good therapeutic targets in other chronic inflammatory pain conditions. The two distinct subtypes of TNF-α receptors have different functions in inflammation. TNFR1 functions mainly in the process that leads to hyperalgesia and most of TNFR2's effects are involved in immunological responses. While depressing the immunological responses is also a way to reduce inflammation induced hyperalgesia, we have chosen to study neuronal TNFR1 effects in this investigation. Significant increases in TNFR1 are relevant based on behavioral evidence showing that antibody against TNFR1 applied to the epineuria abolishes hyperalgesia caused by chronic constriction nerve injury [14]. During inflammation, more TNF-α is released, and TNFR1 expression in neurons is increasing in proportion to the changes in TNF-α release from peripheral tissue and DRG satellite cells [8,29]. A highly active condition in which ROS, TNF-α and TNFR1 are increasing would cause hypersensitivity of peripheral nerves based on our studies presented here for TRPV1 activation and others in literature.

### PBN blocks capsaicin induced TNFR1 increases

ROS are not only endpoint products of TRPV1 activation but also signaling pathway elements. The activation of many types of signaling pathways by ROS involves the direct modification of membrane lipids/proteins or other elements of signaling pathways described for ROS [37]. The present study shows PBN blocks TNFR1 increases induced by capsaicin. It is of considerable interest that ROS are signaling molecules in the regulation of TNFR1 increase after TRPV1 activation. A similar point was held in a study by Gao *et al. *[38] that ROS is involved in enhancement of NMDA-receptor phosphorylation in animal pain models. Biologically relevant ROS include the superoxide radical (•O_2_^-^), hydroperoxyl radicals (HO_2_^•^), H_2_O_2 _and the hydroxyl radical (•OH). It is not clear at this time which component is acting as the factor regulating the TNFR1 in our experiment. Taking ROS in general, however, ROS play roles either as protein phosphate activators or in activation of transcription factors to increase receptor expression. In our experiment, blocking ROS production also abolished TNFR1 increases. ROS has been shown to mediate TRPV1 and neuropeptide-dependent neurogenic vasodilatation in wild type but not TRPV1 knockout mice [39].

### TRPV1 activation induces increases in ROS production

To determine further if ROS is involved in regulation of TNFR1 increases after TRPV1 activation, we also studied ROS production after TRPV1 activation by capsaicin in the mouse DRG neuronal cultures. We assessed cytosolic oxidant activity with use of CM-H2DCFDA, a redox-sensitive fluorochrome probe to monitor ROS production. CM-H2DCFDA oxidation to DCF is a nonspecific reaction used to monitor generation of biological oxidants that include ROS. It is important to recognize that intracellular conversion of H2DCFDA to DCF occurs in competition with other oxidation reactions. The overall rate of H2DCFDA conversion to DCF therefore reflects a complex, highly dynamic balance between simultaneous rates of oxidant production and oxidant buffering by multiple pathways. We interpret this integrated signal as net oxidant activity under a given condition. The data indicate that ROS production is increased by challenging neurons with capsaicin/resiniferatoxin. The results from TRPV1 deficient mice show no increase in ROS production after capsaicin stimulation. The data for the capsazepine pretreatment experiment also confirmed this finding. These data demonstrate that capsaicin initiates ROS generation through TRPV1 activation. The results are supported in experiments done on synoviocyte cultures showing that ROS production was induced by calcium influxes elicited by TRPV1 activation [40]. It has been established that the cation channel TRPV1 is activated by capsaicin or proton and induces calcium influxes [41]. The effective dose used for activation was selected based on previous studies reporting maximal but non-desensitizing responses [3,42,43]. However, the mechanism of generating calcium influx in response to ROS production increase is still unclear. Significant increases in ROS production do not occur in the present study until 30 minutes since ROS levels in cells are tightly regulated. ROS generation during inflammation has attracted increasing attention in the literature. It is also known that the ROS scavenger used to block ROS production also reverses hypersensitivity [23,44]. While much work has been done *in vivo *in rat, our work in cultured mouse DRG neurons challenging TRPV1 with capsaicin to increase ROS production is similar to *in vivo *microenvironment conditions present during inflammation. The finding that PBN abolished ROS production induced by capsaicin in the study further confirms that elevated ROS production is triggered by TRPV1 activation. Thus, ROS are implicated as important functional and potentially therapeutic targets influencing neurotransmitter responses, inflammatory mediator receptors and neurogenic vasodilatation relevant to treatment of inflammation and pain.

## Conclusion

The present study reveals that TRPV1 activation increases ROS which act as signaling molecules to increase TNFR1 in mouse DRG neurons. Thus, in conditions of injured or inflamed tissues, TRPV1 activation increases of ROS and inflammatory mediator TNF-α receptor content could enhance peripheral nerve sensitization, inflammatory pain and potentially other pathological events.

## Experimental methods

This study was performed in accordance with the *Guide for the Care and Use of Laboratory Animals *published by the National Institutes of Health and was approved by the University of Kentucky Institutional Animal Care and Use Committee.

### Primary DRG neuronal cultures

Male and female TRPV1 knockout or wild type C57BL/6 mice (19 – 20 g, Jackson Lab, as a tissue sharing gift from Dr. Lu-Yuan Lee) were used in these experiments [42]. Mice were anesthetized and the vertebral columns were exposed, removed, and put into the ice-cold dissection solution (NaCl 135, KCl 5, KH_2_PO_4 _2, HEPES 10, MgSO_4 _6, CaCl_2 _1.5, Glucose 10; in mM) bubbled with 100% O_2_. The L4, L5 DRGs were removed from the lumbar spinal roots and placed into the 30 mm dish with ice-cold dissection solution. After connective tissues were removed, DRGs were digested in fresh enzyme solution (10 mg trypsin+25 mg collagenase in 10 ml dissection solution) in 25 cm^2 ^flask saturated with O_2_. After shaking in a warm water bath (speed 150 rpm, 35°C) for 1 hour, 4 ml of culture medium (MEM with FBS, antibiotics) were added to the flask. The cells were transferred into a 15 ml centrifuge tube and spun at 800 rpm for 5 minutes. The supernatant was discarded and the cell pellet was re-suspended with culture medium. DRG cells were plated onto poly-L-Lysine pre-coated 12 mm glass-bottom home made tissue culture dish or 12 mm coverslips in 24 well culture plates (0.2 ml of cell suspension per well). Cultured DRG neurons were maintained in an incubator with 5% CO_2_, 37°C overnight and all experiments performed on the subsequent day.

### Immunofluorescence study

Dissociated DRG neurons were cultured on coverslips in 24-well plates overnight. Cells were stimulated with capsaicin (1 μM) or resiniferatoxin (200 nM) for 30 minutes and then the media was replaced with Dulbecco’s phosphate buffered saline (DPBS) for 90 minutes. In some experiments, ROS scavenger PBN was applied for 90 minutes before capsaicin stimulation, or TRPV1 antagonist capsazepine (10 μM) was administered 10 minutes before capsaicin stimulation. At the end of incubation, cells were washed with 0.1 M phosphate buffered saline (PBS, pH7.4) 3 times × 5 minutes and fixed with 4% paraformaldehyde in 0.1 M PBS for 30 minutes. After washing with PBS, cells were incubated with 3% normal donkey serum in PBS plus 0.02% Triton X-100 for 30 minutes to block non-specific protein binding. Then, cells were incubated overnight with the primary antibody diluted in PBS TritonX-100 plus 1% normal donkey serum at 4°C. The anti-TRPV1 receptor antibody (VR1 P-19, Santa Cruz, and affinity purified goat polyclonal antibody against a peptide mapping near the N-terminus of VR1 of rat origin producing a single band in western blots) was diluted 1:100. The anti-TNFR1 receptor antibody (TNF-R1 H-5, Santa Cruz), a mouse monoclonal antibody raised against amino acids 30–301 mapping within the extracellular domain of TNF-R1 of human origin producing a single band in western blots, was diluted 1:50. After overnight incubation, the coverslips were rinsed in PBS 3 times × 5 minutes and incubated with FITC-donkey-anti-goat antibody, diluted 1:1000 and Texas Red-donkey-anti-mouse antibody, diluted 1:800 in PBS Triton-X plus 1% normal donkey serum for 1 hour at room temperature. For staining controls, primary antibodies were omitted. The coverslips were washed again 3 times × 5 minutes. Thereafter, the coverslips were mounted face down onto slides with mounting medium. Five different areas were picked randomly from each coverslip for pictures. MetaMorph offline software was used for digital image data analysis.

### Determination of ROS production by H2DCFDA fluorescent probe

All experiments were done at room temperature. Cells were kept at room temperature for 30 min before replacing culture medium with DPBS, pH 7.4. On the day of the experiment, the ROS-sensitive fluorescent dye, 5-(and-6)-chloromethyl-2', 7'-dichlorodihydrofluorescein diacetate, acetyl ester (CM-H2DCFDA, Invitrogen) was freshly reconstituted. Cells were pre-loaded with CM-H2DCFDA in DPBS at a final concentration of 4 μM for 30 min. The loading buffer was removed and replaced with DPBS allowing 20 min to 2 h recovery time for cells to exhibit low levels of esterase activity. ROS production was monitored for 30 minutes in the live cell imaging experiment. Cells were stimulated with bath application of capsaicin (1 μM) or resiniferatoxin (200 nM) and in some cases neurons were pretreated 10 minutes with TRPV1 antagonist capsazepine (10 μM) before beginning the recording and capsaicin stimulation. ROS scavenger N-tert-Butyl-α-phenylnitrone (PBN, 2 mM) was administered for 90 minutes before capsaicin stimulation.

The change in fluorescent intensity over time is proportional to DCF accumulation, an index of net oxidant activity in the cytoplasm. Fluorescent imaging was performed using an Eclipse 2000-S microscope (Nikon) equipped with a high sensitivity digital monochrome Cool SNAP ES camera (Photometrics, Tucson, AZ) controlled by MetaMorph software (Molecular Devices, Sunnyvale, CA). DCF was excited at 480 nm and emitted light was collected using a 520 nm long pass filter. The photos were captured under 10 ms exposure at 1 min intervals for 30 min. Emissions from DCFH-free buffer are not detectable under these conditions, and background correction is not required. Photooxidation artifact was controlled by conducting experiments in a darkened laboratory and by standardizing excitation parameters to minimize the cumulative delivered energy. The baseline fluorescent intensity was determined prior to exposing the cells to experimental inducements. MetaMorph offline software (Molecular Devices, Sunnyvale, CA) was used for image data analysis.

### Chemicals

Trypsin, poly-L-Lysine, capsaicin, resiniferatoxin, capsazepine and N-tert-Butyl-α-phenylnitrone were obtained from Sigma-Aldrich (St. Louis, MO). Collagenase was purchased from Roche Applied Science (Indianapolis, IN). 5-(and-6)-chloromethyl-2', 7'-dichlorodihydrofluorescein diacetate, acetyl ester and DPBS were purchased from Invitrogen (Carlsbad, CA). Antibody against TRPV1, antibody against TNFR1, FITC-donkey-anti-goat antibody and Texas Red-donkey-anti-mouse antibody were obtained from Santa Cruz Biotechnology (Santa Cruz, CA). Mounting medium was purchased from Vector Laboratories (Burlingame, CA).

### Statistics

All data were expressed as mean ± SEM. Comparisons among multiple means were analyzed with the one-way ANOVA. Post hoc test was analyzed with the Newman-Keuls Multiple Comparison test. A *P *< 0.05 was considered significant.

## Competing interests

The authors declare that they have no competing interests.

## Authors' contributions

All authors have read and approved the final manuscript. FM, LZ and KNW participated equally in the conception, design, and interpretation of the study. FM and LZ carried out the experiments. FM wrote the first draft of the manuscript and prepared the figures. LZ edited the text and figures. KNW edited the text and figures for the submissions.
